# The validation of low-dose CT scans from the [^18^F]-FDG PET-CT scan to assess skeletal muscle mass in comparison with diagnostic neck CT scans

**DOI:** 10.1007/s00259-023-06117-3

**Published:** 2023-02-13

**Authors:** Aniek T. Zwart, Vitor J. Cavalheiro, Maria J. Lamers, Rudi A. J. O. Dierckx, Geertruida H. de Bock, Gyorgy B. Halmos, Anouk van der Hoorn

**Affiliations:** 1 Department of Epidemiology, University Medical Centre Groningen, PO Box 30 001, 9700 RB Groningen, the Netherlands; 2 Department of Radiology, University Medical Centre Groningen, Groningen, the Netherlands; 3 Department of Otolaryngology and Head and Neck Surgery, University Medical Centre Groningen, Groningen, the Netherlands; 4 University of São Paulo, São Paulo, Brazil

**Keywords:** Head and neck neoplasms, Muscle, skeletal, Sarcopenia, Tomography, X-ray computed, Positron emission tomography-computed tomography, Comparative study

## Abstract

**Purpose:**

Radiologically defined sarcopenia, or a low skeletal muscle index (SMI), is an emerging biomarker for adverse clinical outcomes in head and neck cancer (HNC) patients. Recently, SMI measurements have been validated at the level of the third cervical vertebra (C3) on diagnostic neck CT scans but are not yet validated on low-dose (LD) neck CT scans from the [^18^F]-FDG PET-CT. This hampers SMI analysis in HNC patients without a diagnostic neck CT but with a [^18^F]-FDG PET-CT scan. Therefore, the aim was to study whether (low) SMI based on LD CT scan from [^18^F]-FDG PET-CT is comparable to those derived from diagnostic neck CT scans.

**Methods:**

HNC patients with both diagnostic CT and [^18^F]-FDG PET-CT of the neck were prospectively included into the OncoLifeS data-biobank. Skeletal muscle was retrospectively delineated at the level of the third cervical vertebra (C3), and (low) SMI (cm^2^/m^2^) was calculated for diagnostic and LD neck CTs. (Low) SMI from the diagnostic neck CT was considered the reference standard. Intra-class correlation coefficient (ICC), Bland–Altman plots, and Cohen’s Kappa analysis were performed.

**Results:**

The cohort (*n* = 233) mean age was 66.2 ± 12.8 years, and 74.2% of patients were male. Inter-rater reliability was excellent (ICC > 0.990, 95% confidence interval 0.975–0.996, *p* < 0.001). The agreement of SMI between both modalities was high according to the Bland–Altman plot (mean ΔSMI =  − 0.19 cm^2^/m^2^), and there was no substantial bias. Cohen’s Kappa analysis showed an almost perfect agreement of low SMI between the two modalities (*κ* = 0.911, *p* < 0.001). The position of arms didn't affect the high agreement of (low) SMI.

**Conclusion:**

Skeletal muscle mass, as measured with (low) SMI, remains constant irrespective of CT acquisition parameters (diagnostic neck CT scans versus LD neck scans of the [18F]-FDG PET-CT scan), positioning of arms, and observers. These findings contribute to the construction of a clinically useful radiological biomarker for SMI and therefore identify patients at risk for adverse clinical outcomes.

## Introduction

Annually, head and neck cancer (HNC) is responsible for almost 900,000 cases and 450,000 deaths worldwide [1]. More than 90% of cancers of the head and neck region are squamous cell carcinoma [2], with approximately two-thirds of newly diagnosed HNC patients having advanced stage disease [2, 3]. Moreover, patients with HNC are predisposed to malnutrition, which is most pronounced in patients with advanced disease [4]. Recently, low skeletal muscle mass is a phenotypic criterion for malnutrition and should be the focus of nutritional interventions, according to the Global Leadership Initiative on Malnutrition [5]. Even more importantly, low skeletal muscle mass on imaging, also known as radiological sarcopenia, is emerging as a radiological biomarker for frailty and adverse clinical outcome in HNC patients [6–9].

Single slice cross-sectional area (CSA, cm^2^) measurements of the abdominal musculature at the third lumbar vertebra (L3) are the gold standard to calculate the skeletal muscle index (SMI, cm^2^/m^2^), and the latter is seen as a surrogate marker for total body skeletal muscle mass [10, 11]. As a result of lacking abdominal imaging in HNC patients, Swartz et al. demonstrate that it is feasible to predict CSA at L3 by using the CSA of neck musculature at the third cervical vertebra (C3), and using that input to calculate the SMI of the patient [12]. CSA measurements at C3 were further validated for MRI [13], making skeletal muscle mass assessment at C3 level a multi-modality biomarker. In current literature, two main imaging groups are used to calculate SMI on CT: diagnostic neck CT and low-dose (LD) CT usually from the [18F]-FDG PET-CT. However, the use of LD CT to measure SMI based on C3 CSA has not been validated yet.

In general, [^18^F]-FDG PET-CT imaging in HNC is helpful for pre-treatment oncological staging, selection, and delineation of target volume for radiotherapy planning, treatment response assessment, and post-therapy follow-up. Validation of SMI calculation using LD CT at C3 level benefits patients receiving a [^18^F]-FDG PET-CT but have no diagnostic neck CT or MRI imaging. That would allow the assessment of skeletal muscle mass and in return evaluate the patient’s risk for malnutrition and adverse clinical outcome. Furthermore, validation of LD CT at C3 level to calculate SMI provides the possibility to monitor skeletal muscle mass during follow-up in cases with [^18^F]-FDG PET-CT imaging and could play a role in the prevention or treatment of muscle wasting in the case of nutritional or physical interventions. Therefore, the aim was to study whether SMIs and radiological sarcopenia diagnosis (low SMI) based on LD CT scans from the [^18^F]-FDG PET-CT scan are comparable to those derived from diagnostic neck CT scans, the current reference standard in HNC patients. This present study hypothesised that the assessment of skeletal muscle mass, as measured with SMI and radiologically defined sarcopenia, can be reliably measured on the LD neck CT scan from the [^18^F]-FDG PET-CT.

## Materials and methods

### Study design and medical ethical approval

This was a retrospective cohort study using prospectivelly gathered data from OncoLifeS, which is a large-scale institutional oncological data-biobank from the Netherlands, approved by the Medical Ethical Committee of the University Medical Center Groningen (UMCG) [14]. Access to the data for the here presented study was approved by the scientific board of the UMCG. Patients with pathologically proven cancer were enrolled in the OncoLifeS data-biobank after written informed consent was secured, and the following details were collected and stored: clinical and treatment data, comorbidities, lifestyle, radiological and pathological findings, biomaterials, quality of life, and long-term outcomes [14].

### Study population and data collection

**Fig. 1:**
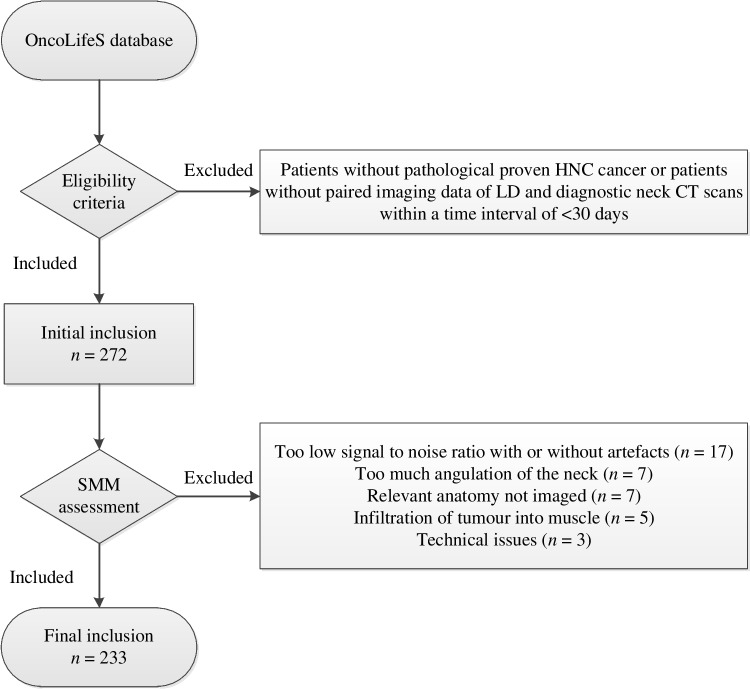
Flow diagram of included patients. HNC, head and neck cancer; LD, low dose; SMM, skeletal muscle mass

Inclusion criteria were patients with pathologically proven mucosal or cutaneous HNC cancer, irrespective of oncological stage, with paired data of diagnostic neck CT scans and the LD CT scan from the [^18^F]-FDG PET-CT. The maximal time frame between the two modalities was 30 days or less, and it was not mandatory that imaging was made at baseline. Between August 2011 and March 2019, 272 patients were identified for the initial sample size. Patients were further excluded (*n* = 39, 14.3%) due to several reasons including the following: too low signal-to-noise ratio with or without artefacts (*n* = 17), patients with severe kyphosis or other cervical deformities resulting in the presence of multiple vertebrae on the axial slice at the level of C3 (*n* = 7), relevant anatomy not imaged (*n* = 7), no differentiation of muscle possibly due to muscle infiltration of the tumour (*n* = 5), and technical issues (*n* = 3). The final inclusion was 233 patients (see Fig. 1 for the flowchart).

Collected data were as follows: sex, age (years), weight (kg), height (m), body mass index (BMI, kg/m^2^), tumour site, oncological stage according to the seventh edition of the Union for International Cancer Control TNM Classification, and treatment modality. Included patients were treated according to the applicable national guidelines which were deliberated by the multidisciplinary head and neck tumour board.

### Image acquisition parameters

In this present study, all imaging used for analysis were made during the normal oncological work-up. Diagnostic neck CT was considered the reference standard, as image quality is superior to LD CT. Manufacturers responsible for diagnostic neck CT imaging were as follows: Biograph 64 (*n* = 171), SOMATOM Definition AS (*n* = 39), Biograph 40 (*n* = 12), SOMATOM Force (*n* = 5), Biograph 128 Edge (*n* = 2), Sensation Open (*n* = 2), SOMATOM Definition Flash (*n* = 2), and SOMATOM Definition (*n* = 1). Acquisition parameters for diagnostic CT scans were as follows: 80–140 kV, 512 × 512 matrix, 500 mm field of view (FOV), 1.0–2.0 mm slice thickness, 0.5–1.0 spacing, soft tissue reconstruction (B30f, I26s, BR40, B20s), and use of intravenous iodine contrast in 217 (93.1%) of cases.

 LD CTs acquired during the [^18^F]-FDG PET-CT imaging were generated by Biograph 64 (*n* = 215), Biograph 40 (*n* = 16), and Biograph 128 Edge (*n* = 2) CT scanners. No intravenous iodine contrast was used in LD CT imaging, and other acquisition parameters for LD CT scans were as follows: 100–140 kV, 512 × 512 matrix, 500 mm FOV, 3.0 mm slice thickness, 1.5 spacing, and soft tissue reconstruction kernel (B30f). CT dose index volume was 4.93 ± 0.72 mGy and 0.44 ± 0.43 mGy for diagnostic and LD CT scans, respectively. Another apparent difference between the two modalities was the positioning of the arms. On diagnostic CT imaging, all patients had their arms down. On the LD neck CT, most patients also had their arms down (*n* = 200), but 31 patients had their arms up. Therefore, the outcome of this present study was also stratified for positioning of the arms based on the LD neck CT.

### Skeletal muscle mass analysis and radiologically defined sarcopenia

**Fig. 2:**
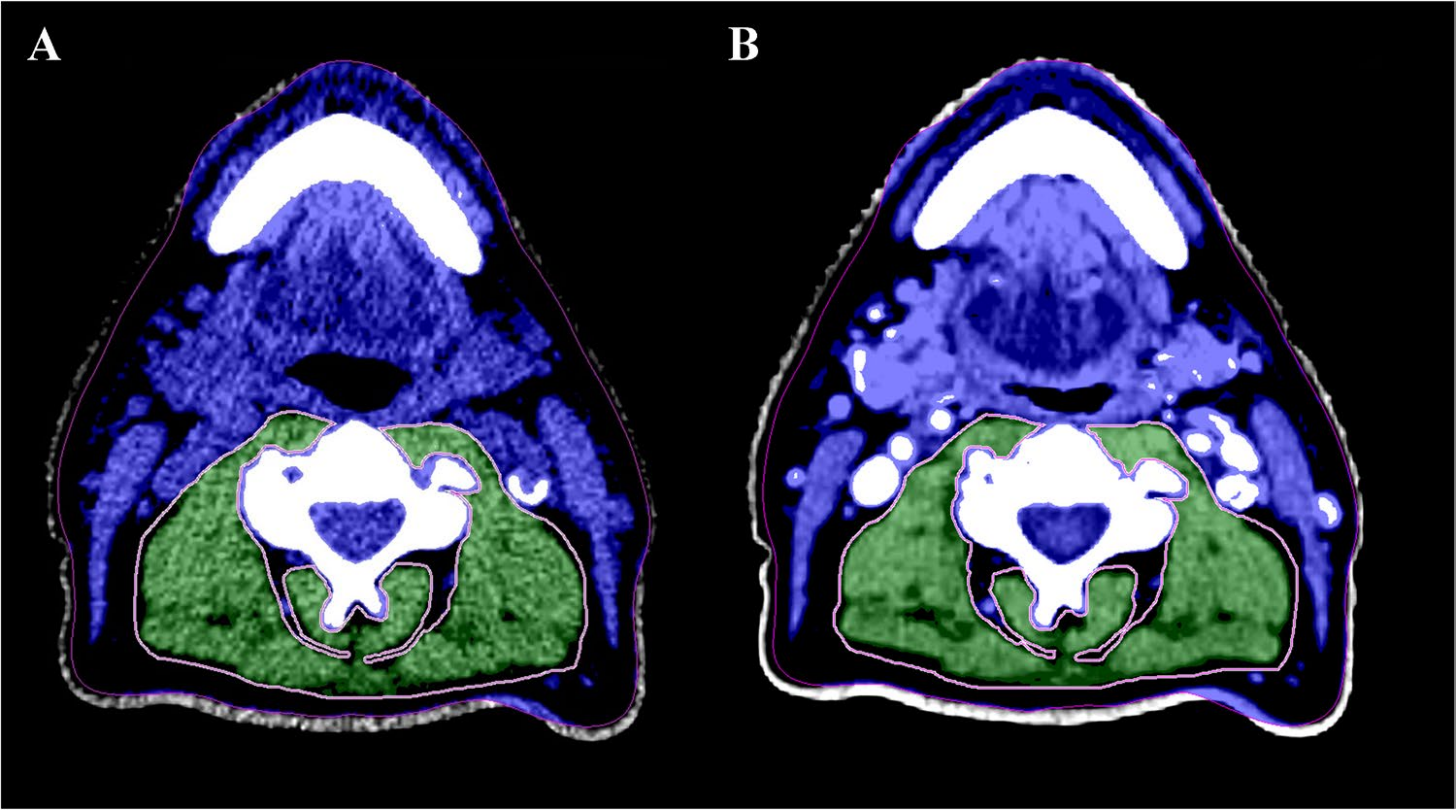
Delineation of paravertebral muscle in green at C3 level made on LD from the [^18^F]-FDG PET-CT scan (**A**) and diagnostic neck (**B**) CT scan. Blue and green are within the skeletal muscle HU thresholds. Additionally, both sternocleidomastoid muscles were delineated to calculate the CSA at C3 level. C3, third cervical vertebra; CSA*,* cross-sectional area; CT, computed tomography; LD, low dose

All measurements were conducted in a semi-automatic way using the Aquarius workstation iNtuition edition program (Ver.4.13.P6, TeraRecon Inc.). The validated procedure of Swartz et al. was used to perform skeletal muscle analysis. Scrolling from the caudal to cranial direction, the first slice with a closed vertebral arch of C3 was selected [12]. Hounsfield units (HU) threshold were set to − 29 until + 150 HU which corresponds with muscle density [11], and thus automatically excluding all other densities within the delineation. Total CSA (cm^2^) at C3 was the added CSA of the paravertebral and both sternocleidomastoid muscles (see Fig. 2 showing an example of paravertebral muscle delineation on LD and diagnostic neck CT scan). CSA at L3 level was then calculated with the equation of Swartz et al. and afterward corrected for patient stature (m^2^) resulting in the patient SMI (cm^2^/m^2^) (see Eqs. 1 and 2, respectively) [12]. To define radiologically defined sarcopenia, or low SMI, sex-specific SMI cut-off values were applied. The present study aimed to select previously published SMI cut-off values with a similar cohort of HNC patients based on age, ethnicity, and cancer site and stage. Eventually, the sex-specific SMI cut-off values of van Rijn-Dekker et al. and Zwart et al. were selected [15, 16]. 

### Observer reliability

The main observer (V.J. Cavalheiro, observer 1) was a medical student who performed all measurements in this present study, with the use of a step-by-step manual. CSA measurements at C3 level were first conducted in ten diagnostic CT scans, and inter-observer reliability was tested against other observers (PhD student (A.T. Zwart, observer 2) with 4 years experience doing these measurements, and three other medical students). After training, observer 1 performed the measurement in the real dataset under the supervision of observer 2. Additionally, the inter-observer reliability of the main observer was tested for the measurements made in the real dataset but then against a board-certified radiologist specialised in head and neck imaging (M.J. Lamers, observer 3); this for twenty randomly selected diagnostic and LD CT neck scans. Observers who conducted measurements for the observer analysis were blinded by each other outcomes.

1 $${\mathrm{CSA at L}3 (\mathrm{cm}}^{2})=27.304+1.363*\mathrm{CSA at C}3 \left({\mathrm{cm}}^{2}\right)-0.671*\mathrm{Age }\left(\mathrm{years}\right)+0.640*\mathrm{Weight }\left(\mathrm{kg}\right)+26.442*\mathrm{Sex }(1=\mathrm{Female},2=\mathrm{Male})$$

2 $$\mathrm{SMI }({\mathrm{cm}}^{2}/{\mathrm{m}}^{2})={\mathrm{CSA at L}3 (\mathrm{cm}}^{2})/\mathrm{Height }({\mathrm{m}}^{2})$$

### Statistical analysis

Continuous characteristics were tested for normality with the Kolmogorov–Smirnov analysis and Q–Q plots and presented as mean with standard deviation (SD) or median with interquartile range (IQR), for respectively normal and non-normal distributed characteristics. Categorical characteristics were presented with frequencies and percentages of the total. Inter-observer reliability was measured with an intra-class correlation coefficient (ICC) and provided with 95% confidence intervals (CI). For the first part of the research aim, the agreement of SMI between the two modalities, a Bland–Altman plot was generated including mean SMI (diagnostic neck CT + LD neck CT / 2), ΔSMI (diagnostic neck CT − LD neck CT), and limits of agreement (LOA) with 1.96SD and − 1.96SD. A linear regression was performed to evaluate potential proportional bias and was plotted into the Bland–Altman plot in case of a significant bias. To evaluate the agreement in radiologically defined sarcopenia diagnosis as the second part of the research aim, two different sets of SMI cut-off values [15, 16] were applied to diagnose low SMI, and a Cohen’s Kappa (*κ*) analysis was conducted. An almost perfect agreement of diagnosis was considered if *κ* > 0.81. Furthermore, the Bland–Altman plot and Cohen’s Kappa analysis were stratified for positioning of arms, to evaluate if positioning of arms affects the agreement of SMI and sarcopenia diagnosis. The intended sample size was determined including as many eligible patients in the analysis. *α* was < 0.05. SPSS version 25 (IBM, Armonk, NY) was used for statistical analyses.

## Results

### Baseline characteristics of the cohort

Of the included cohort, the mean age was 66.2 ± 12.8 years, the median BMI was 24.3, IQR was 7.2 kg/m^2^, and about three-quarters of patients (74.2%) were male. The most common tumour site was the oropharynx (39.9%) followed by the larynx (34.3%), hypopharynx (9.9%), oral cavity or lip (5.6%), cutaneous (4.3%), nasopharynx (3.4%), and unknown primary (2.6%) (see Table 1 for the other baseline characteristics of included patients).

**Table 1:** Baseline characteristics of included patients

	Total *n* = 233
Sex	
Female	60 (25.8%)
Male	173 (74.2%)
Age (years)	66.2 ± 12.8
BMI (kg/m^2^)	24.3 IQR 7.2
Tumour site	
Oropharynx	93 (39.9%)
Larynx	80 (34.3%)
Hypopharynx	23 (9.9%)
Oral cavity or lip	13 (5.6%)
Cutaneous*	10 (4.3%)
Nasopharynx	8 (3.4%)
Unknown primary	6 (2.6%)
Oncologic stage**	
I	5 (2.2%)
II	22 (9.5%)
III	53 (22.9%)
IV	151 (65.4%)
Missing	2
Treatment modality	
(Chemo-)radiation	197 (84.5%)
Surgery	33 (14.2%)
Deceased before initiating therapy	2 (0.9%)
Palliative chemotherapy	1 (0.4%)

Categorical data is given with percentage of total group size *n*. Continuous data is given as mean with standard deviation or as median with interquartile range (IQR)

*BMI* body mass index

^*^Histology of cutaneous-located malignancies was squamous cell carcinoma (*n* = 5), melanoma (*n* = 3), angiosarcoma (*n* = 1) and pleomorphic dermal sarcoma (*n* = 1)

^**^Staging confirmed with the 7th edition of American Joint Committee on Cancer Manual

### Observer reliability

The performance of the main observer in the sample (*n* = 10) with diagnostic neck CT scans, which was used for teaching purposes, was excellent (ICC = 0.998, 95% CI 0.995–0.999, *p* < 0.001). Observer analysis was repeated in twenty randomly selected patients from the real sample size, and the performance of the main observer remained excellent in diagnostic (ICC = 0.992, 95% CI 0.980–0.997, *p* < 0.001) and LD (ICC = 0.990, 95% CI 0.975–0.996, *p* < 0.001) neck CT scans.

### Agreement of SMI between LD neck and diagnostic CT scans

**Fig. 3:**
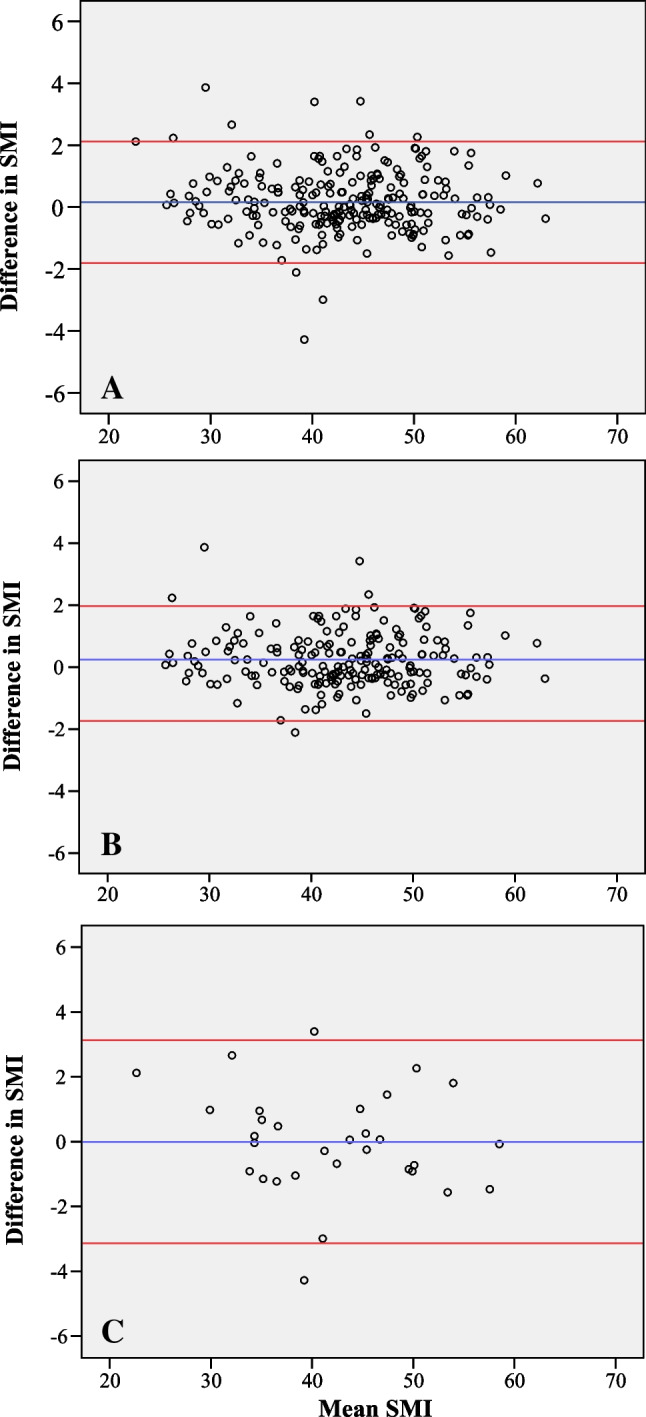
Bland–Altman plots showing the agreement of SMI as measured at the level of the third cervical vertebra between LD CT scans from the [^18^F]-FDG PET-CT scan and diagnostic neck CT scans for total cohort (**A**), patients with their arms having down on the LD CT (**B**), and patients with their arms having up on the LD CT (**C**). The difference in SMI = diagnostic neck CT − LD neck CT. Mean SMI = diagnostic neck CT + LD neck CT / 2. The blue line is the mean SMI difference, and the red lines are limits of agreement with + 1.96 SD and − 1.96 SD. CT, computed tomography; LD, low dose; SMI, skeletal muscle index

Most diagnostic and LD CT scans were made on the same day (*n* = 196), and the median was 5.0 (IQR 11.0) days between the LD and the diagnostic CT scan. The mean SMI for the total cohort was 43.27 ± 7.71 cm^2^/m^2^ and 43.08 ± 7.77 cm^2^/m^2^ for diagnostic and low-dose neck CT scans, respectively. For the total cohort, no significant proportional bias could be identified for SMI calculated with LD and diagnostic neck CT scans, as indicated by the linear regression analysis (*p* = 0.405). The mean difference of SMI of both modalities was − 0.19 cm^2^/m^2^, with limits of agreement between − 1.78 and 2.16 cm^2^/m^2^ (see the Bland–Altman plot (Fig. 3A)). In total, 10 (4.29%) outliers outside of limits of agreement could be identified.

The Bland–Altman plots stratified for positioning of arms on the LD neck CT scan showed also a high agreement independent of positions of arms (Figs. 3B and C). There was no proportional bias on the LD CT scan according to the linear regression analysis (*p* = 0.356 and *p* = 0.786 for the group with arms up and down, respectively). However, agreement of SMI between the two modalities performed slightly better in the case of arms down on the LD CT scan, as there was fewer variance in SMI difference according to the limit of agreement and fewer outliers (2.50%, *n* = 5 vs. 6.45%, *n* = 2) compared to the patients having their arms up. Although minimal, the mean difference of SMI was better in the group having their arms up (− 0.004 cm^2^/m^2^) compared to the group with their arms down (0.88 cm^2^/m^2^) on the LD CT scan.

### Agreement of sarcopenia diagnosis between LD and diagnostic neck CT scans

As mentioned previously, two sets of SMI cut-off values were applied to diagnose low SMI or radiologically defined sarcopenia. Based on the SMI cut-off values of Zwart et al., 60.5% and 57.9% of patients were found to be sarcopenic in the diagnostic CT and LD CT groups, respectively. Agreement of low SMI was almost perfect between the two modalities (*κ* = 0.929, *p* < 0.001) and was not affected by the positioning of the arms with contradicting positioning of the arms (*n* = 31, *κ* = 1.000, *p* < 0.00) and similar position of the arms (*n* = 200, *κ* = 0.917, *p* < 0.001).

Similar results were found when applying the SMI cut-off values of van Rijn-Dekker et al. The prevalence of radiologically defined sarcopenia according to van Rijn-Dekker et al.’s SMI cut-off values was 42.1% and 40.3% for diagnostic and LD neck CT scans, respectively. Agreement in sarcopenia diagnosis between the two modalities was almost perfect (*κ* = 0.911, *p* < 0.001). According to Cohen’s Kappa analysis, agreement of sarcopenia diagnosis was similar in patients having contradicting positioning of arms (down on diagnostic neck CT versus up on LD neck CT) compared to patients with corresponding positioning of arms (down on both scan types) resulting with *κ* = 0.936, *p* < 0.001 and *κ* = 0.906, *p* < 0.001, respectively.

## Discussion

This present study found excellent inter-observer reliability for SMI as measured with CSA at C3 level for both LD and diagnostic CT neck imaging. Moreover, a high agreement of SMI and radiologically defined sarcopenia diagnosis (low SMI) between LD and diagnostic neck CT was observed. Positioning of arms did not affect the agreement in (low) SMI between the two modalities.

To our knowledge, this was the first study to analyse the agreement of SMI and radiologically defined sarcopenia as derived from CSA measurements at C3 level on LD neck CTs from the [^18^F]-FDG PET-CT, using diagnostic neck CTs as reference. Swartz et al. generated a formula to calculate the CSA at L3 based on the CSA of C3 corrected for patient characteristics and found a high correlation (*r* = 0.891, *p* < 0.001). They studied a small group of 52 HNC patients who received a whole-body FDG-PET (low-dose) CT and 51 patients without HNC who underwent a total-body (diagnostic) CT in a trauma setting. It could be hypothesised that the correlation of CSA at C3 and L3 is equal within these two imaging groups because of paired imaging data. This could be true but is not guaranteed as, e.g., low-dose imaging could impair the signal-to-noise ratio in abdominal imaging more than neck imaging, since abdominal imaging has more volume. Our study added to this previous study showing a good correlation of SMI based on C3 CSA between the diagnostic and low-dose CT in a large group of HNC patients, which has not been previously shown. Although Swartz et al. does not compare SMI outcomes at C3 level on both imaging modalities, others found similar results of SMI outcomes at L3 as measured with different CT acquisitions parameters [17, 18]. The phantom study of Kim et al. analysed the reliability of CSA measurements at L3 level using various CT acquisition parameters (e.g. CT manufacturers, radiation dose, slice thickness, and image reconstruction algorithms) and found a good similarity in CSA for all acquisition parameters compared to the standard protocol [17]. Moreover, the study of van Vugt et al. showed reliable identification of low SMI at L3 level using different intravenous iodine contrast phases [18]. The results of the study of van Vugt et al. are in line with the results of this present study, showing a good overlap of SMI between LD CT and contrast-enhanced diagnostic neck CT. The effect of positioning of the arms on SMI calculation was not previously published for skeletal muscle measurements made at C3 level. van Heusden et al. analysed the correlation of CSA between the fourth thoracic vertebra (Th4) and L3 and identified that positioning of the arms negatively impacted the correlation, mostly due to variance of the pectoralis muscle CSA [19]. In the contrary, the present study showed that positioning of the arms had minimal effect on the agreement of C3 SMI between LD and diagnostic neck CT. This difference in outcome is mainly explained by the insertion of the arms to the upper body, where the placement of the arms probably does not cause a lot of movement of the neck musculature, but does affect the pectoralis muscle. Although the published literature is limited, the results of the present study seem to be supported by the previous studies of van Vugt et al. and Kim et al., supporting the main results that the assessment of SMI at C3 level is reliable irrespective of heterogeneous CT acquisition parameters and positioning of the arms.

Some limitations should be addressed. First, there is no consensus on SMI cut-off values to diagnose low SMI, and this could be seen as a limitation. Therefore, we selected the two sets of sex-specific SMI cut-off values from previously published literature with a similar cohort of heterogeneous HNC patients. Irrespective of applied SMI cut-off values, this present study showed an almost perfect agreement of low SMI between LD and diagnostic CT, indicating that the agreement of low SMI between LD and diagnostic CT is good, and it is not affected by the applied SMI cut-off value. Second, heterogeneity of applied CT acquisition parameters existed within the diagnostic neck CTs in the present study. Ideally, CT acquisition parameters in diagnostic neck CTs were more homogenous, but the high agreement between LD and diagnostic neck CT scans implies that CT acquisition parameters do not influence SMI. Third, there also was heterogeneity in the study population, including multiple locations and oncological stages of HNC that may have influenced the outcome, although it should be mentioned that HNC in itself represents a heterogeneous oncological group.

The latter, however, could also be seen as a strength as these two heterogeneities resemble daily practice, thus making our study more transferable to clinical practice. Another strength is the observer reliability analysis that was performed for the main observer in the sample for training purposes and in the real sample as well, showing excellent performance of measurements at C3 level on both LD and diagnostic CT neck scans. Furthermore, most of the LD and diagnostic CT scans were made on the same day which limits the risk for possible pathophysiological changes of skeletal muscle between two measurements. Moreover, the present study had a relatively large prospectively included cohort of HNC patients for validation, with respect to other validation studies. Future research should be focused on the clinical value of adding low muscle strength or function to the sarcopenia diagnosis in HNC patients, on the external validation of SMI cut-off values in HNC and on interventional studies (nutrition and/or exercise) to maintain skeletal muscle mass during treatment which hopefully reduces adverse events.

## Conclusions

This present study found that skeletal muscle mass, as measured with (low) SMI using the CSA at C3 level, remains constant irrespective of CT acquisition parameters (diagnostic neck CT scans versus LD neck scans of the [^18^F]-FDG PET-CT scan), positioning of arms, and observers. This is important as skeletal muscle mass status in HNC patients is related to frailty, malnutrition, and adverse clinical outcomes [6–9].

## Data Availability

The datasets generated and/or analysed during the current study are not publicly available due to privacy restrictions but are available from the corresponding author on reasonable request.

## Abbreviations

BMI: Body mass index
C3: Third cervical vertebra
CI: Confidence intervals
CSA: Cross-sectional area
HNC: Head and neck cancer
HU: Hounsfield unit
ICC: Intra-class correlation coefficient
IQR: Interquartile range
SD: Standard deviation
SMI: Skeletal muscle index
SMM: Skeletal muscle mass
L3: Third lumbar vertebra
LD: Low dose
LOA: Limits of agreement
OR: Odds ratio
